# Implantation of an extravascular implantable cardioverter-defibrillator in a patient with retrosternal adhesions from epicardial leads implanted via a subxiphoid approach

**DOI:** 10.1016/j.hrcr.2026.06.037

**Published:** 2026-07-03

**Authors:** Michael Douglas, Akshaya Gadre, Todd Crawford, John D. Serfas, Amit Noheria, Seth H. Sheldon

**Affiliations:** 1 Department of Cardiovascular Medicine, The University of Kansas Health System, Kansas City, Kansas; 2 Department of Cardiovascular Surgery, The University of Kansas Health System, Kansas City, Kansas

**Keywords:** Implantable cardioverter-defibrillator, Extravascular, Epicardial, Cardiac surgery, Congenital heart disease, Complete heart block

**Key Teaching Points**

• A multi-disciplinary implant attempt in conjunction with cardiac surgery may be used for extravascular (EV)-implantable cardioverter-defibrillator (ICD) implantation in patients with retrosternal adhesions secondary to abandoned epicardial pacing leads and limited alternative options for ICD implantation.
• Preprocedural imaging and multidisciplinary collaboration, including cardiac surgery, are necessary in such patients.
• The precise procedural risk of EV-ICD implantation in the presence of retrosternal adhesions remains uncertain.

## Introduction

Extravascular (EV) implantable cardioverter-defibrillator (ICD) is distinct from transvenous and subcutaneous ICD types in that its implantation is extracardiac but intrathoracic, using a substernal position. EV-ICD is contraindicated in patients with prior sternotomy, as the resultant retrosternal adhesions pose risks to mediastinal structures during the tunneling for placement of the lead.^1^ However, the implantation risks of EV-ICD in collaboration with cardiac surgery may be justifiable in select patients with retrosternal adhesions when alternative options are disadvantageous. We present a case of subxiphoid EV-ICD implantation in a patient with systemic ventricular dysfunction, easily inducible ventricular fibrillation (VF) on electrophysiology (EP) study, congenital complete heart block (cCHB), and retrosternal adhesions secondary to abandoned epicardial pacemaker leads.

## Case report

A 27-year-old man with complex congenital heart disease including a history of situs inversus totalis, mesocardia, congenitally corrected transposition of the great arteries (ccTGA), status post transcatheter closure of atrial septal defect, and cCHB presented with an elevated shock coil impedance consistent with shock coil fracture. He was pacing dependent with a cardiac resynchronization therapy (CRT-D) and had abandoned epicardial pacing leads placed in infancy. The original epicardial pacemaker was placed via a subxiphoid approach without sternotomy. He was converted to a transvenous system at age 10 years with removal of the subrectus pacemaker generator and capping of the epicardial leads. At age 16 years, his transvenous system was extracted with plans to reimplant a dual-chamber pacemaker due to concern that his growth and limited lead redundancy presented an imminent threat of ventricular pacing lead malfunction. During the procedure, he had 5 episodes of VF, often occurring with only 1 or 2 ventricular ectopic beats. After multi-disciplinary discussion and involvement of his family, it was elected to proceed with dual-chamber ICD implantation.

**Figure 1:**
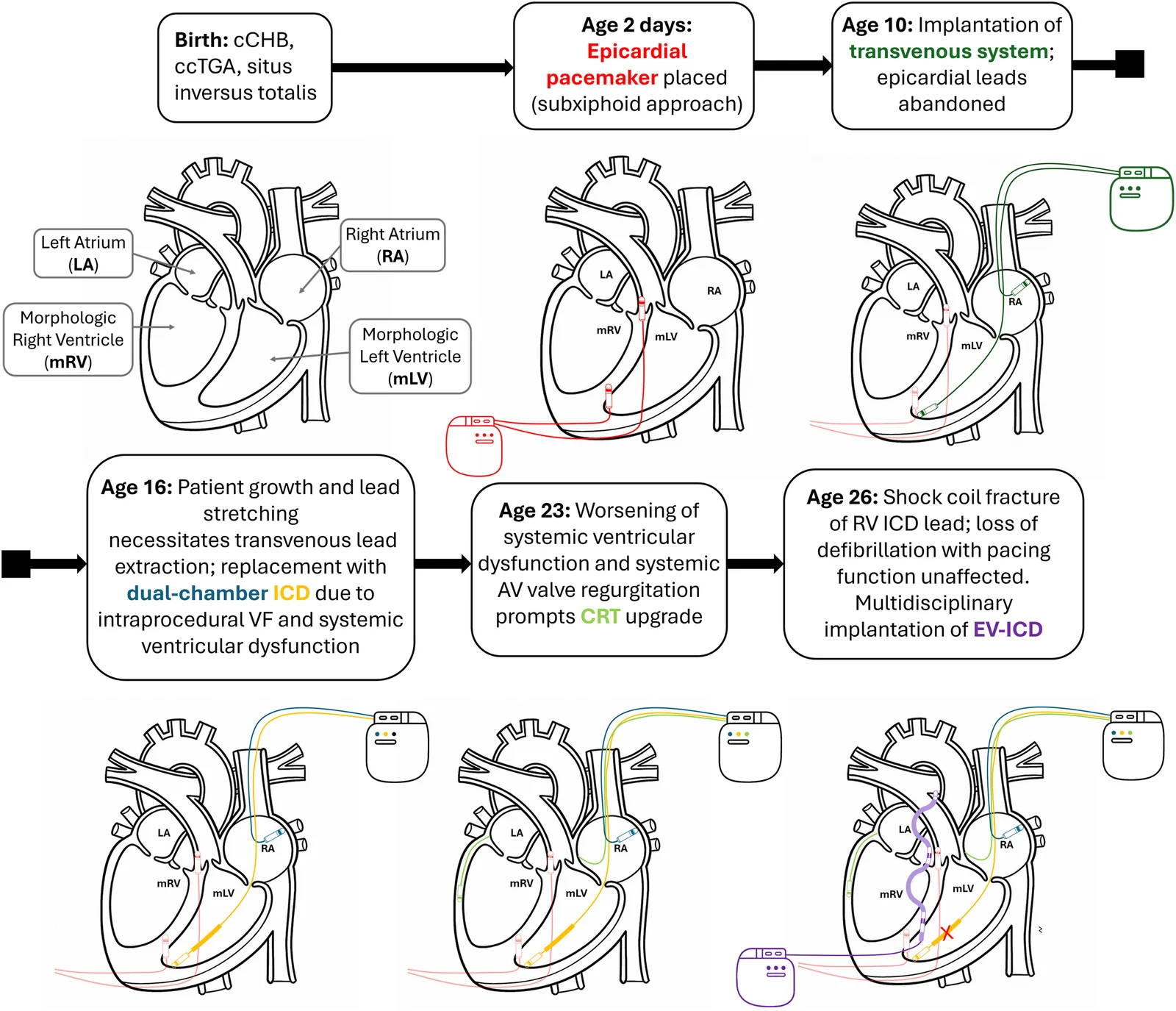
Chronological outline of device history. Flowchart timeline outlining the patient’s congenital heart disease background and cardiac device history, illustrating the patient’s progression from epicardial pacemaker utilization in infancy to EV-ICD implantation in young adulthood. AV = atrioventricular; cCHB = congenital complete heart block; ccTGA = congenitally corrected transposition of the great arteries; CRT = cardiac resynchronization therapy; EV-ICD = extravascular implantable cardioverter-defibrillator; ICD = implantable cardioverter-defibrillator; RV = right ventricular; VF = ventricular fibrillation.

His systemic ventricular function continued to deteriorate (visually estimated systemic ventricular ejection fraction 30%–35% on echocardiogram and likely lower in the setting of severe systemic AV valve regurgitation). Accordingly, he underwent CRT-D upgrade at age 24 years, during which the coronary sinus lead was ultimately placed in the superior branch of a large lateral systemic ventricular vein. His systemic ventricular function subsequently improved, qualitatively described as low-normal, and his systemic AV valve regurgitation improved from severe to mild according to a 1-year post-CRT echocardiogram. The patient’s cardiac and device history are summarized in Figure 1.

At age 26 years, he developed an elevated right ventricular (RV) shock coil impedance consistent with shock coil fracture. His tip and ring electrode parameters remained normal with no impairment in RV pacing function. His defibrillator detections/therapies were deactivated. There had been no instances of cardiac arrest or appropriate ventricular arrhythmia therapies since the ICD was placed, but the longitudinal risk of sudden cardiac death remained elevated due to systemic ventricular dysfunction (which at this point was qualitatively described by echocardiogram as moderately reduced) and severe systemic ventricular hypertrophy/dilation as observed on cardiac computed tomography angiography/echo. Furthermore, he demonstrated a propensity towards easily inducible VF at his prior device procedure. Although the possibility of continuing without an ICD was discussed, the patient strongly preferred to retain an ICD.

A multi-disciplinary committee discussed options. The prospect of extraction and replacement of the fractured RV ICD lead, although ideal, had several concerns: (1) the high risk of dislodging the coronary sinus lead in part due to recent placement and lead–lead binding and interaction—the placement of this lead was quite difficult given the patient’s unusual anatomy and involved intraprocedural dislodgement and repositioning; (2) the high risk of extraction failure given the marked tortuosity of the ICD lead and the position of the ICD coil against the walls of the heart; (3) risk of damaging the sub-pulmonary mitral valve apparatus as the redundancy of the atrial lead was seen protruding in to the valve plane; (4) risk of paradoxical embolus from a residual right-to-left shunt despite the transcatheter atrial septal defect closure. For those reasons, extraction/replacement was not recommended. He was screened for subcutaneous ICD placement but was ultimately not a candidate.

**Figure 2:**
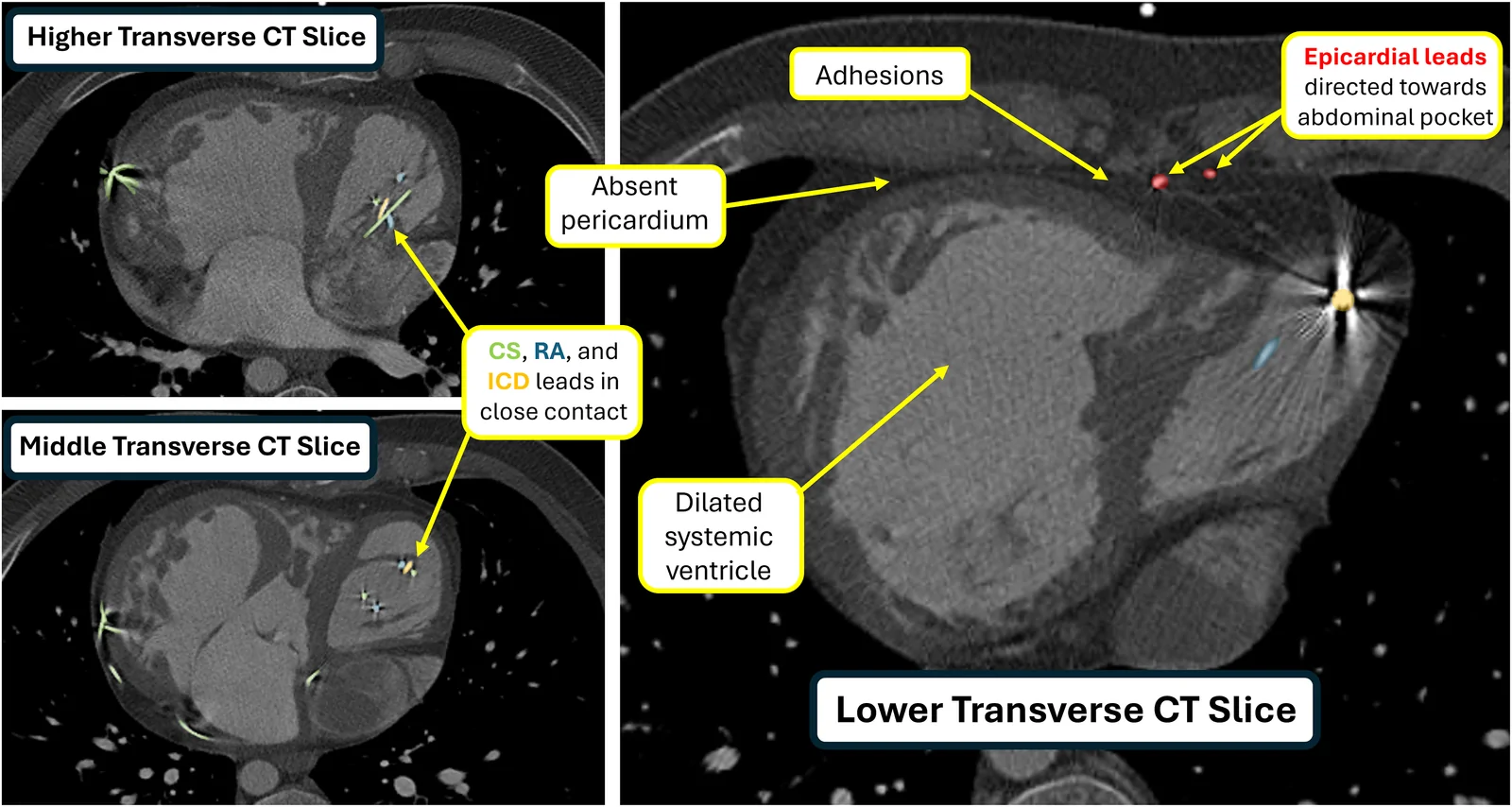
Pre-procedural computed tomography (CT) Scan. High, middle, and low slices from a preprocedural computed tomography scan, illustrating the course of abandoned epicardial leads, the presence and location of retrosternal adhesions, absence of the pericardium in the low retrosternal space, and close proximity of the coronary sinus, right atrial, and implantable cardioverter-defibrillator (ICD) leads. These findings informed risk assessment of management options and preprocedural planning for extravascular (EV)-ICD implantation. CS = coronary sinus; EV = extravascular; ICD = implantable cardioverter-defibrillator; RA = right atrial.

Alternatively, he was felt to be a reasonable candidate for EV-ICD placement with a collaborative approach between cardiac surgery and electrophysiology. His abandoned epicardial leads had resulted in retrosternal adhesions visible on chest computed tomography (Figure 2). At the multidisciplinary conference, the risks of EV-ICD placement were estimated to be less than those of extraction/reimplantation or continuing without defibrillator protection. While acknowledging the possibility of future pacing failure of his ICD lead, it was recommended to proceed with EV-ICD.

The patient was taken to a hybrid suite with cardiac surgery and electrophysiology operators. After general anesthesia induction, a transverse subxiphoid incision was made. The incision was then extended in a “hockey stick” shape cephalad over a preexisting lower midline incision. The fascial attachments to the xiphoid process were dissected around its inferior border to fully mobilize it. At this point, dense adhesions from his abandoned epicardial leads were encountered and bluntly lysed with finger dissection. Cephalad progression through the substernal space was achieved with additional finger dissection along the posterior table of the sternum.

**Figure 3:**
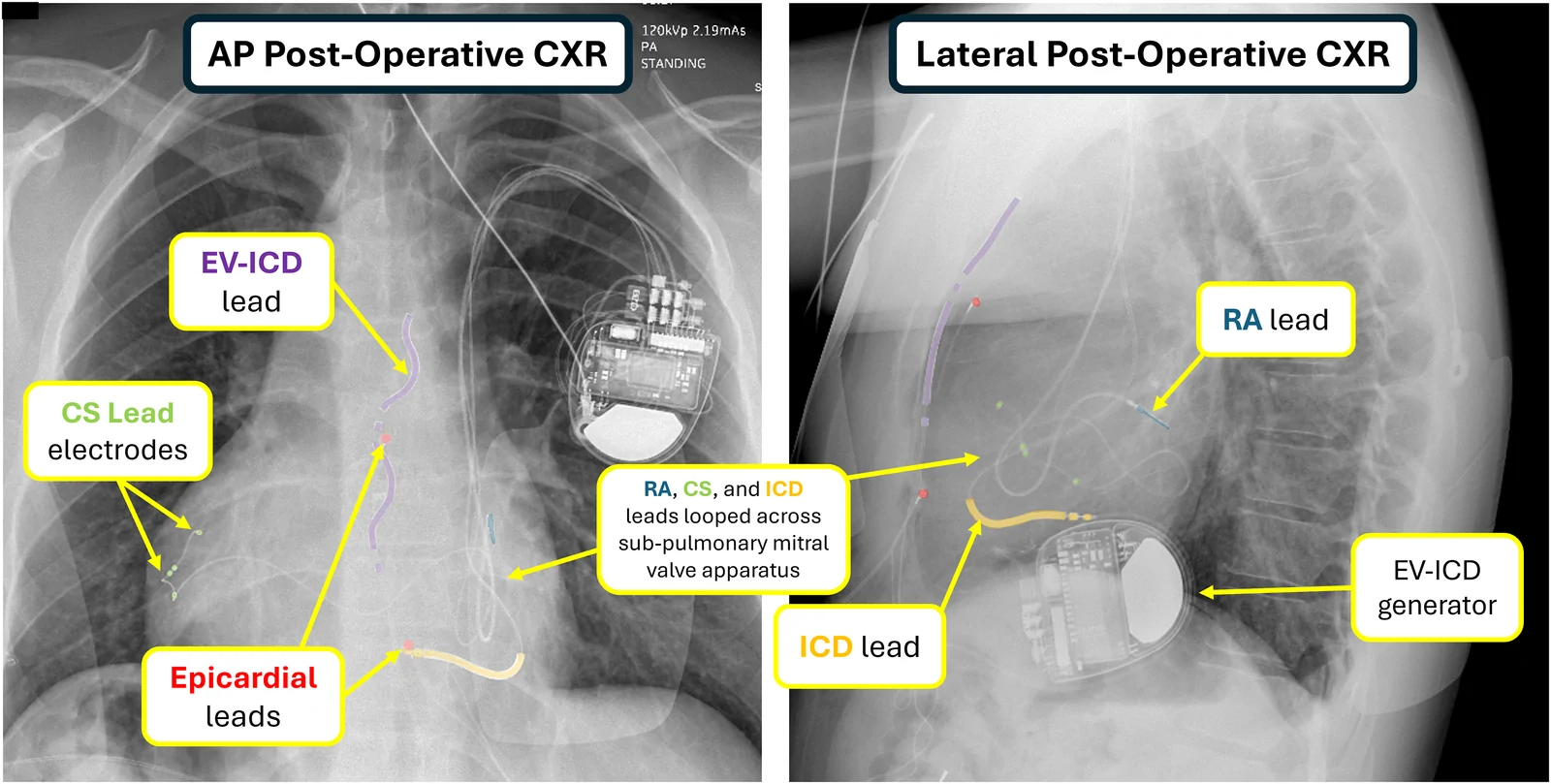
Postprocedural anteroposterior and lateral chest X-rays. Postprocedural anteroposterior and lateral chest X-rays demonstrating final positioning of EV-ICD lead relative to the abandoned epicardial pacing leads and other preexisting hardware. Findings also illustrate elements of previous risk assessment regarding extraction and replacement of the RV ICD lead. CS = coronary sinus; EV-ICD = extravascular implantable cardioverter-defibrillator; ICD = implantable cardioverter-defibrillator; RA = right atrial.

Using lateral fluoroscopy, the tunneling rod was then inserted into the sub-sternal space, contacting the posterior aspect of the xiphisternal junction. The tunneling tool was advanced superiorly to the top of the cardiac silhouette. The ICD lead was then advanced through the sheath. Testing demonstrated R waves on ring 1 to ring 2 of 5 mV with P-waves 0.2 mV or less. A horizontal right submammary incision was made to create an intermuscular pocket to house the generator. The transverse tunneling tool was used to create a subcutaneous tract from the subxiphoid incision over the costal rib margin to the device pocket incision. The lead was then pulled through to the device pocket and connected to the generator. Postoperative chest radiography demonstrated the final EV-ICD lead and generator position relative to the existing pacing system (Figure 3).

Defibrillation testing was performed using 50 Hz induction of VF. The patient was successfully internally defibrillated with 25.8 Joules (coils-to-can defibrillation vector; maximum ICD output of 40J) and remained hemodynamically stable after defibrillation testing. There was no appreciable interaction between the epicardial pacing leads and the EV-ICD lead. The patient tolerated the procedure well without immediate complications. Upon 3-month follow-up, the patient was feeling well and denied any functional limitations.

## Discussion

Care for patients with adult congenital heart disease often necessitates a multi-disciplinary, collaborative approach, and consideration of non-conventional treatments. The EV-ICD is contraindicated in patients with significant sub-sternal adhesions due to prior sternotomy. We present a patient with a history of prior epicardial pacing leads placed via a sub-xiphoid approach who underwent EV-ICD implantation in a combined procedure with cardiac surgery and electrophysiology. We are not aware of other instances of EV-ICD implantation in patients with epicardial pacing leads.

Exclusion criteria for EV-ICD safety and efficacy trials included any prior medical condition or procedure that leads to adhesions in the anterior mediastinal space, such as prior mediastinal instrumentation.^2^ Such adhesions can impair the advancement of the substernal tunneling tool and pose major risks to mediastinal structures, including cardiac perforation and damage to the mediastinal vasculature. As the presence of such adhesions resulted in exclusion from safety and efficacy trials, the true procedural risk in light of such adhesions remains uncertain. Although our patient underwent successful EV-ICD implantation, this was performed by experienced operators with involvement of a cardiac surgeon. This case is presented not to promote widespread implantation of EV-ICD in patients with prior sub-sternal instrumentation/adhesions, but rather to demonstrate its feasibility with a collaborative approach in select patients without optimal alternative options.

Preprocedural chest imaging is helpful to assess the anatomy before EV-ICD implantation. This is especially critical before EV-ICD implantation in a patient with suspected substernal adhesions. Imaging can be used to identify the location of adhesions and whether or not a subxiphoid approach may reach an area without appreciable adhesions as a starting point for placing the tunneling tool and initiating tunneling. It can also highlight the proximity of the cardiac border to the sternum, location of sternal wires (if present), and the optimal location for tunneling.

There are reports of EV-ICD implantation in patients at risk for substernal adhesions. EV-ICD implantation has been successfully achieved in a patient with prior mitral valve surgery via a right-sided thoracotomy (access to cardiac structures via a more posterolateral right-sided pericardial incision while avoiding anterior pericardial access) as well as in a patient with prior sternotomy and residual sternal wires.^3^^,^^4^ A collaborative approach between electrophysiology and cardiac surgery is helpful for these cases, and preprocedural imaging is critical. Alternatively, an open cardiac surgical approach could be used for EV-ICD implantation in such patients, as has been described by Deets et al.^5^ This is more invasive and introduces uncertainty in device/lead testing at the time of implantation.

The use of an EV-ICD in a patient with epicardial pacing wires has the potential to result in lead–lead interaction. We implanted the EV-ICD lead in proximity to 1 of the epicardial pacing leads but with some separation seen on the lateral CXR (Figure 3). No appreciable lead–lead interaction/noise was seen in the initial 3 months post-implant. Defibrillation testing was also successful at implant.

The selected approach in our patient does not overcome the risk of future pace/sense failure from his fractured RV ICD lead. It may be necessary to consider further options in the future if/when this occurs. This may require implanting a lower-profile pacing lead. Alternatively, conduction system leadless pacing may be an option when this occurs, for which the proof-of-principle has recently been demonstrated by Reddy et al, using a novel helix-based leadless pacemaker.^6^

## Conclusion

Our case demonstrates the feasibility of safe implantation of EV-ICD in patients with retrosternal adhesions from prior abandoned epicardial pacing leads. EV-ICD implantation in select patients with potential retrosternal adhesions should only be considered after careful review of preprocedural imaging and utilization of a collaborative approach including cardiac surgery.

## Disclosures

S Sheldon: Abbott (honoraria for educational conference), Biosense Webster (consulting), Boston Scientific (honoraria for educational conference), Medtronic (honoraria for educational conference and consulting). The other authors have no other disclosures to report.
